# Supplementary material for: Probing the function of Streptomyces albidoflavus J1074 gene XNR_5296 for SPOUT family ribose methyltransferase
Source: Microbiol Spectr. 2025 Nov 26;14(1):e02192-25. doi: 10.1128/spectrum.02192-25 (PMC12772374; doi:10.1128/spectrum.02192-25)
Supplement: Supplemental material — Tables S1 to S3; Fig. S1 to S7. [file spectrum.02192-25-s0001.pdf]

Electronic Supplemental Material for the paper of Tseduliak *et al*. Probing the function of *Streptomyces albidoflavus* J1074 gene *XNR_5296* for SPOUT family ribose methyltransferase

Inventory of Supplemental Material

Supplemental Tables, Figures and Legends

Table S1. Nucleosides from *S. albidoflavus* J1074 and Δ5296 total tRNA samples detected by LC-MS/MS
Table S2. Plasmids and strains used in this work
Table S3. Primers used in this work
Fig. S1. LC-MS/MS data on modified nucleoside 2-thio-2'-O-methyluridine (**s2Um**) in comparison with s2Um standard
Fig. S2. LC-MS/MS data on modified nucleoside 2-lysidine (**k^2C**)
Fig. S3. LC-MS/MS data on modified nucleoside 4-acetylcytidine (**ac^4C**).
Fig. S4. Alignment of Xnr_5296 and experimentally verified TrmJs
Fig. S5. Alignment of Xnr_5296 and experimentally verified TrmLs
Fig. S6. Structural comparison of AlphaFold predicted structure of Xnr_5296 and (A) TrmJs, (B) TrmLs and (C) rRNA methyltransferases
Fig. S7. Generation of the Δ5296 strain

Table S1. Nucleosides from *S. albidoflavus* J1074 and Δ5296 total tRNA samples detected by LC-MS/MS

Nucleoside	MH[+1]	NLS[2]	Rt – std[3]	Rt – obs[4]	J1074	Δ5296
**D**, dihydrouridine	247	132	3.4	3.5	+	+
**Ψ**, pseudouridine	245	36	3.8	3.7	+	+
**m^{1}A**, 1-methyladenosine	282	132	7.6	7.0	+	+
**m^{7}G**, 7-methylguanosine	298	132	7.4	8.5	+	+
**k^{2}C**, 2-lysidine	372	132	9.5	7.7	+	+
**m^{5}C**, 5-methylcytidine	258	132	7.6	7.7	+	+
**Cm**, 2'-*O*-methylcytidine	258	146	9.4	7.9	+	+
**I**, inosine	269	132	9.9	9.0	+	+
**Um**, 2'-*O*-methyluridine	259	146	10.6	10.8	+	-
**m^{5}Cm**, 5,2'-*O*-dimethylcytidine	272	146	11.7	11.7	+	+
**s^{4}U**, 4-thiouridine	261	132	13.5	12.5	+	+
**m^{1}G**, 1-methylguanosine	298	132	13.4	13.4	+	+
**Gm**, 2'-*O*-methylguanosine	298	146	13.8	13.8	+	+
**ac^{4}C**, 4-acetylcytidine	286	132	14.0	13.2	+	+
**s^{2}Um**, 2-thio-2'-*O*-methyluridine	275	146	15.5	15.5	+	+
**t^{6}A**, N^6-threonylcarbamoyladenosine	413	132	18.6	15.6	+	+
**m^{6}A**, N^6-methyladenosine	282	132	21.0	20.0	+	+
**ms^{2}A**, 2-methylthioadenosine	314	132	23.8	24.2	+	+
**io^{6}A**, N^6-(cis hydroxyisopentenyl) adenosine	352	132	28.1	27.8	+	+
**ms^{2}io^{6}A**, 2-methylthio- N^6-(cis hydroxyisopentenyl) adenosine	398	132	34.0	33.8	+	+
**i^{6}A**, N^6-isopentenyladenosine	336	132	34.6	34.4	+	+
**ms^{2}i^{6}A**, 2-methylthio- N^6-isopentenyladenosine	382	132	37.8	37.8	+	+

[1] Cation m/z
[2] Fragmentation pattern of the nucleoside, e.g. either loss of ribose (132 Da) or 2'-O-methylated ribose (146 Da), or pseudouridine dissociation (36 Da)
[3] Retention time of the nucleoside standard, wherever available (–, absence of the standard)
[4] Retention time for the presumed modified nucleoside observed in this work

Nucleosides highlighted in grey are tentatively considered for the first time to be present, or reliably detected (s^{2}Um), in total tRNA hydrolyzates from *S. albidoflavus* strains.

Table S2. Plasmids and strains used in this work

Plasmid/strain	Description	Source*
pKC1132	Am^r; suicidal Streptomyces vector	(48)
pTES	Ingetrative expression vector; ermEp, aac(3)IV, phiC31	
patt-shyg	Ap^r, Hy^r; contains a synthetic hygR6Kγ, flanked with B-CC and P-GG sites for φC31 integrase	(49)
pKC5296KO	Amr; pKC1132 carrying 3-kb fragment with *XNR_5296* cloned into HindIII/EcoRI sites	This work
pKC5296KOhyg	Am^r, Hy^r; pKC5296KO, with ∆XNR_5296::hyg replacement (hyg is from patt-shyg)	This work
pTES5296	pTES carrying *XNR_5296* cloned as XbaI-EcoRI fragment under ermEp*	This work
E. coli ET12567 pUZ8002	Cm^r, Km^r; dam-13::Tn9 dcm-6 hsdM; harbors conjugative plasmid pUZ8002	C.P. Smith, UMIST, UK
E. coli BW25133 pKD46	Ap^r; host for recombineering experiments; harbors plasmid pKD46 with λ red genes	(40)
S. albidoflavus SAM2	*S. albidoflavus* J1074 derivative; deletion of pseudo φC31 site (pseB4)	(44)
S. albidoflavus ∆5296	Hy^r; ΔXNR_5296::hyg mutant	This work
S. albidoflavus CS5296	Am^r; complementation strain (∆5296, carrying the pTES5296 plasmid)	This work
Bacillus cereus ATCC 19637	Test culture to detect antibacterial compounds	ATCC
Debaryomyces hansenii Y-9	Test culture to detect antifungal compounds	MCCAP https://mccap.org.ua/

Hy^r; hygromycin resistant; Am^r, apramycin resistant; Kmr, kanamycin resistant; Cm^r, chloramphenicol resistant.
*Numbers in the brackets refer to the Reference list in the main text

Table S3. Primers used in this work

Name	Sequence, 5´ → 3´	Purpose
xnr5296_dup	AAAAAGCTTCTGGTGCGTACGCGCCGAC	To generate pKC5296KOhyg
xnr5296_drp	AAAGAATTCGTCCCAGCCAGCAGACGATTC	
trmL_red_up	CCTGACGCGACCGCCACCGAACGGGAGCCC GCACCCCATGTTCCGGGGATCCGTCGACC	To generate pKC5296KOhyg
trmL_red_rp	GAGACCGGGACGGCGGGGCCCGTCCGGAAC GCGGTGCTCATGTAGGCTGGAGCTGCTTCG	
xnr5296_XbaIup	AAATCTAGACATCGTCATCGGCGAGGTCG	To clone *XNR_5296*
xnr5296_EcoRI_rp	AAAGAATTCGTCCGGAACGCGGTGCTCAG	
hyg_diagn2	CCACGTGGTGCCCGTCATG	To verify the knockout (together with xnr5296_XbaIup

Sites for restriction endonucleases are underlined in the primer sequence

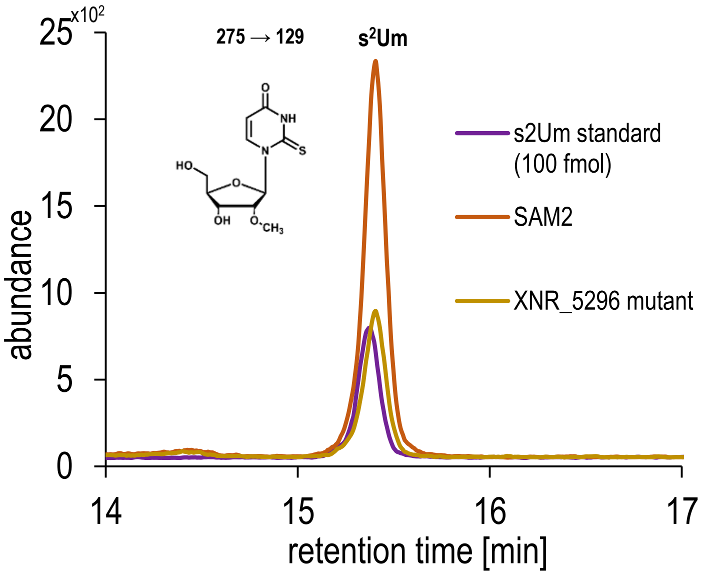

Fig. S1. Detection of the 2-thio-2'-O-methyluridine (s^{2}Um) modification by LC-MS/MS in tRNA samples isolated from *S. albidoflavus* SAM2 and XNR_5296 mutant. An overlay of the extracted ion chromatograms at a mass transition of m/z 275 → 129 obtained from QQQ LC-MS/MS analysis of the digested SAM2 tRNA and digested XNR_5296 tRNA in comparison with s2Um standard (100 fmol injected).

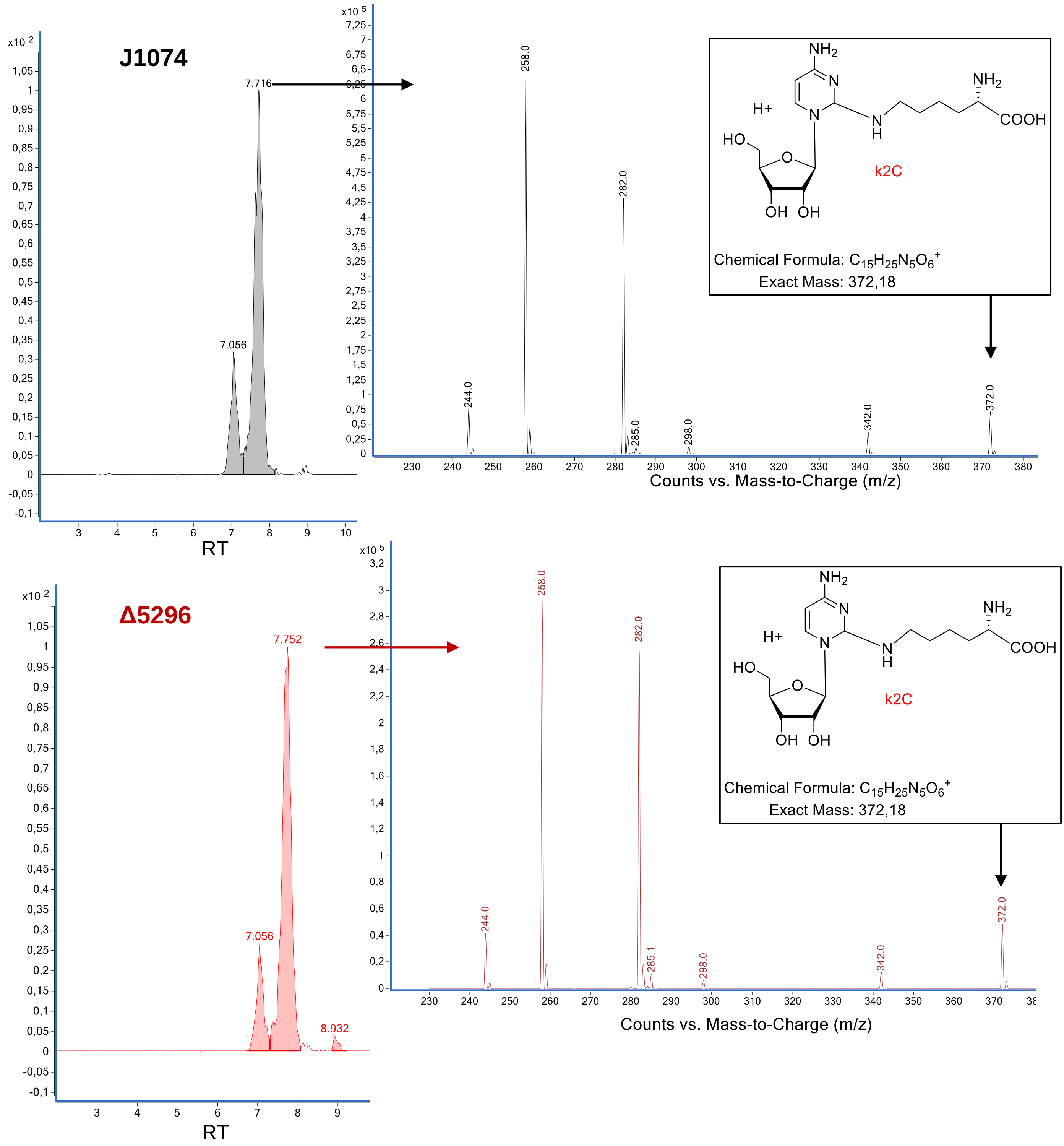

Fig. S2. LC-MS/MS data on modified nucleoside 2-lysidine (k^2C). Top, EIC (NLS) for k^2C from wild type J1074 strain and MS spectrum data for specified RT. Bottom, EIC (NLS) for k^2C from mutant Δ5296 strain and MS spectrum data for specified RT.

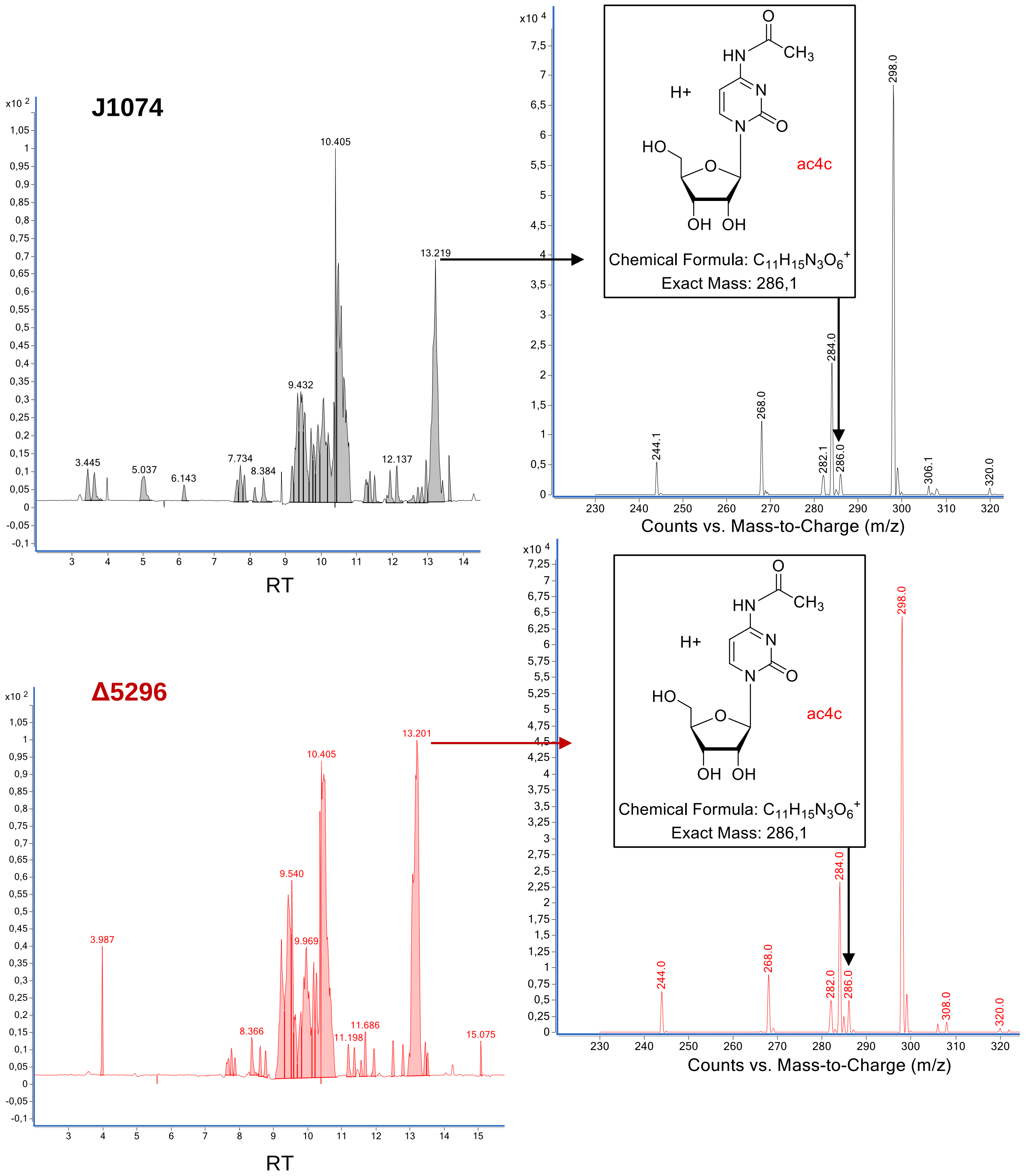

Fig. S3. LC-MS/MS data on modified nucleoside 4-acetylcytidine (ac4C). Top, EIC (NLS) for ac4C from wild type J1074 strain and MS spectrum data for specified RT. Bottom, EIC (NLS) for ac4C from mutant Δ5296 strain and MS spectrum data for specified RT.

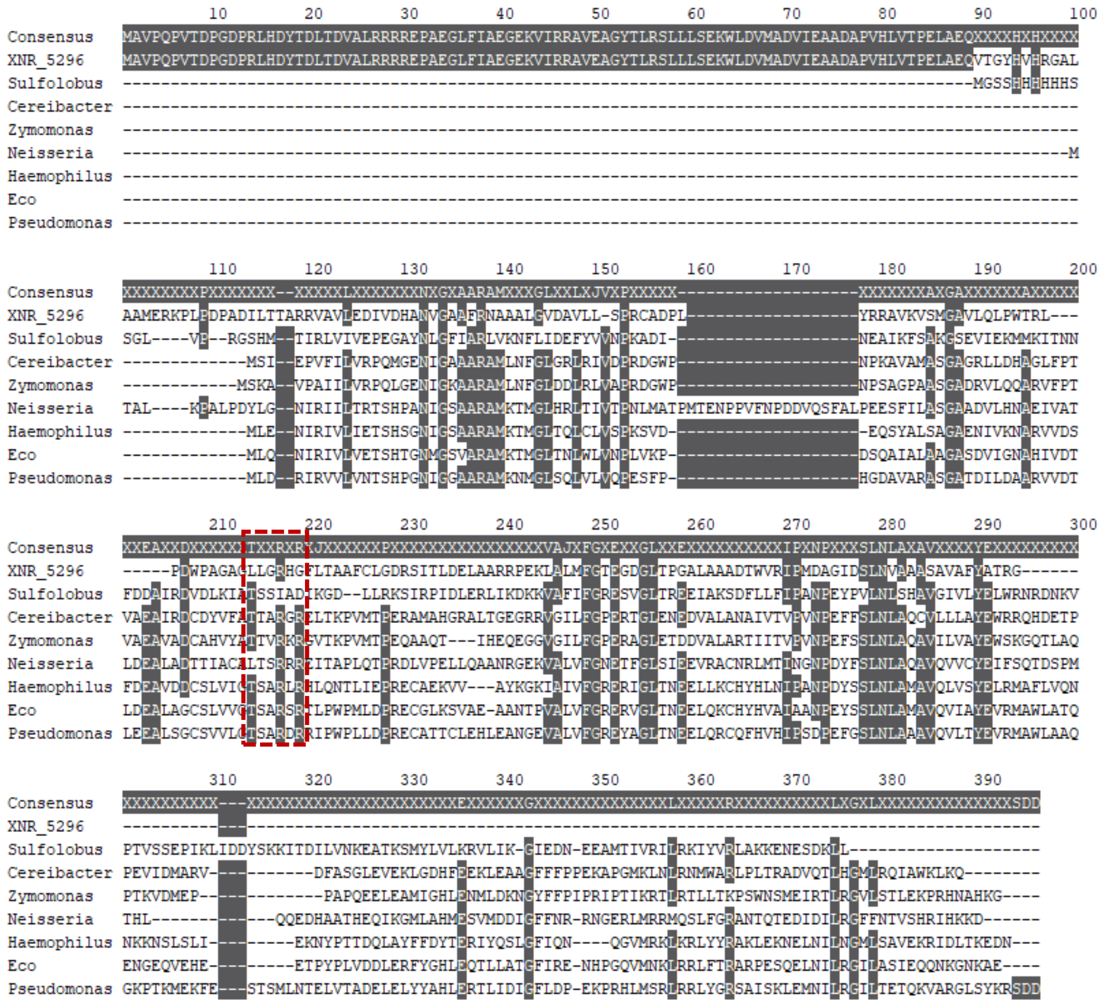

Fig. S4. Alignment of XNR_5296 and experimentally verified TrmJs: *Sulfolobus acidocaldarius* (PDB id 4CND), *Cereibacter sphaeroides* (3ONP), *Zymomonas mobilis* (5GRA), *Neisseria gonorrhoeae* (3IC6), *Haemophilus influenzae* (3ILK), *Escherichia coli* (4XBO), *Pseudomonas aeruginosa* (5GMC). The red dotted line indicates TrmJ-specific motif (36). Alignment generated using Clustal Omega algorithm in Geneious Prime 2025.1.2 software.

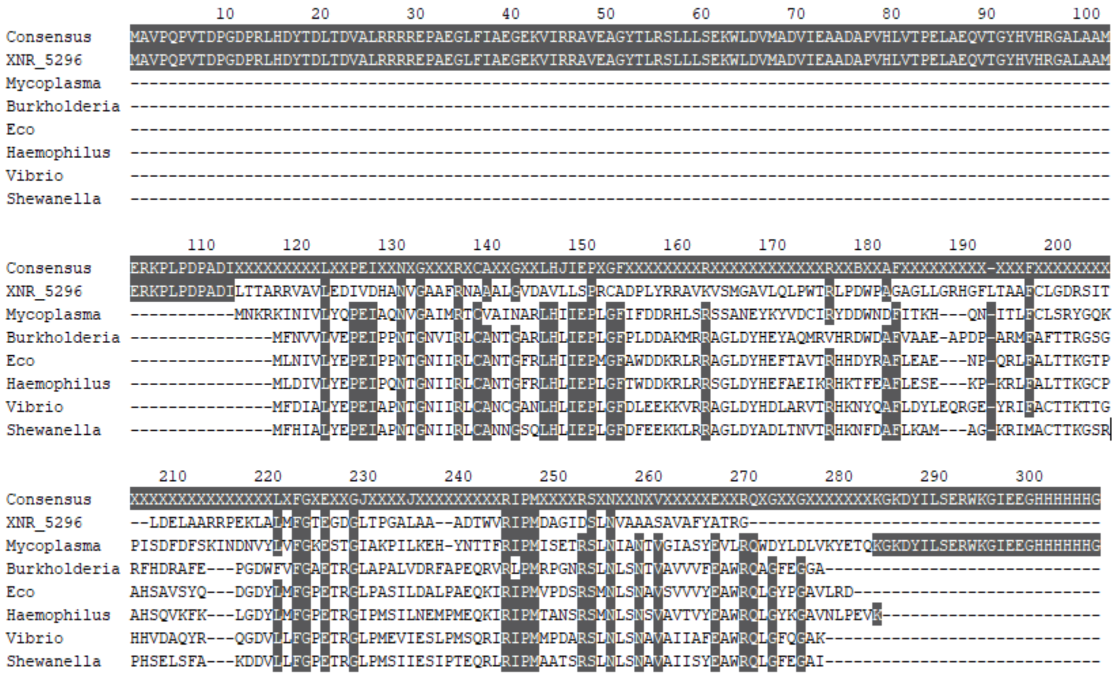

Fig. S5. Alignment of XNR_5296 and experimentally verified TrmLs: *Mycoplasma capricolum* (PDB id 7E3T), *Burkholderia pseudomallei* (4KGN), *Escherichia coli* (4KDZ), *Haemophilus influenzae* (7D51), *Vibrio vulnificus* (7E3Q), *Shewanella oneidensis* (7E3S). Alignment generated using Clustal Omega algorithm in Geneious Prime 2025.1.2 software.

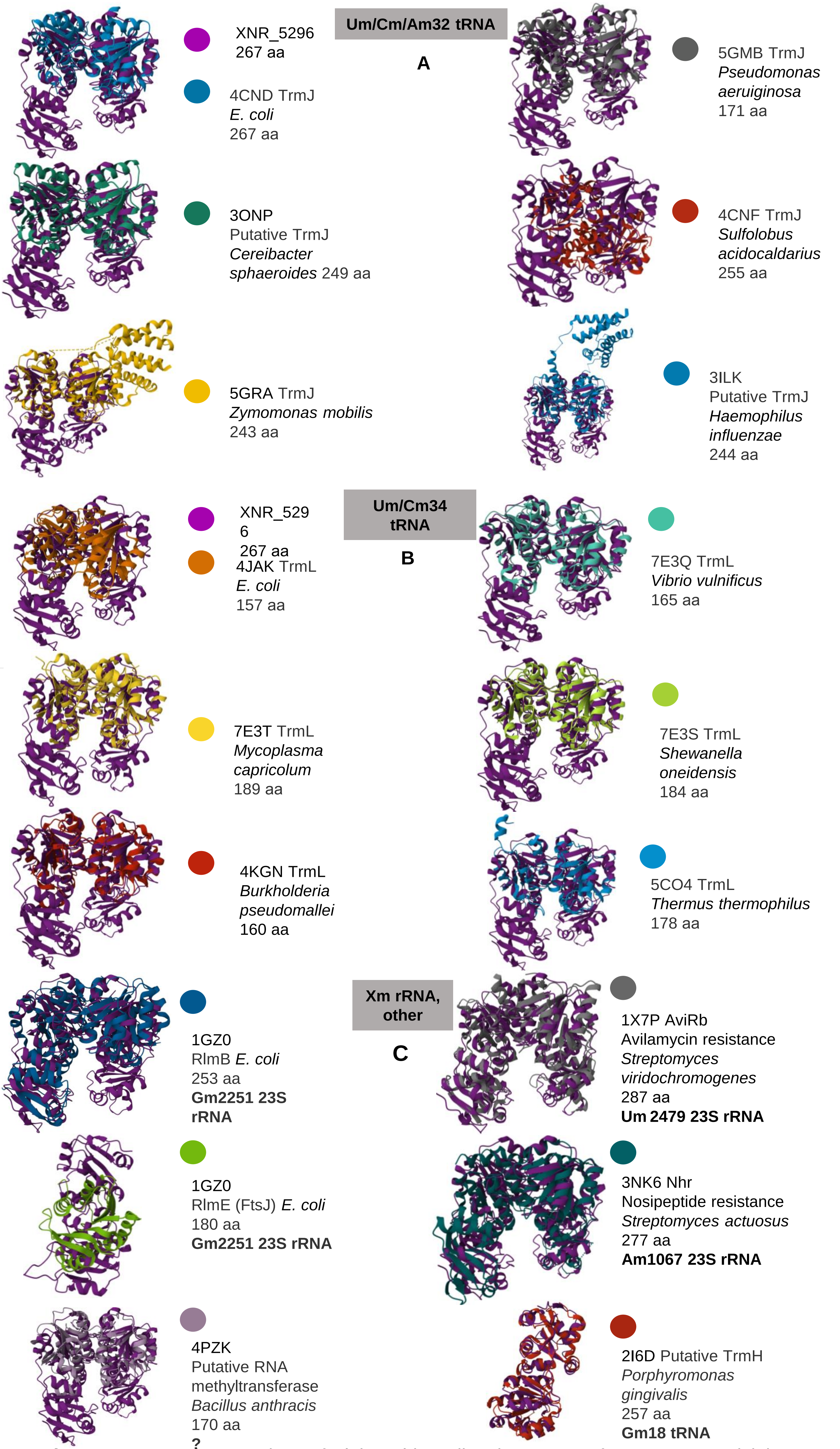

Fig. S6. Structural comparison of AlphaFold predicted structure of XNR_5296 and (A) TrmJs, (B) TrmLs and (C) rRNA methyltransferases. Model superposition was carried out at https://www.rcsb.org/3d-view.

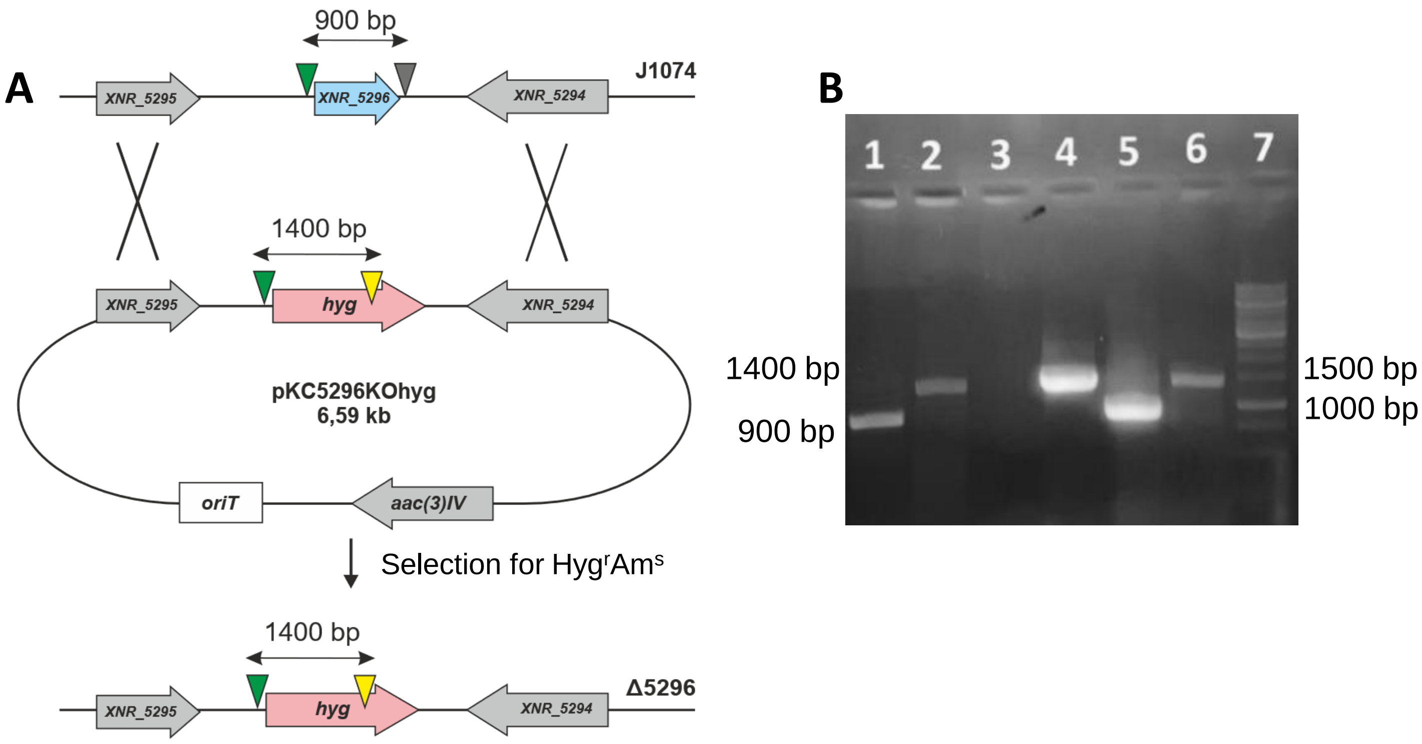

Fig. S7. Generation of the Δ5296 strain: (A) Replacement of XNR_5296 with hyg marker scheme. Colored triangles mark primers used in PCR: green for Xnr1636_XbaIup, grey for Xnr1636_XbaIup and yellow for hyg_diagn_2 (primer sequences are listed in Table S3), (B) PCR verification of XNR_5296 replacement: 1 – J1074 genome DNA (900 bp), 2 - Δ5296 genome DNA (1400 bp), 3 - negative PCR control (without DNA), 4 - pKC5296KOhyg plasmid (1400 bp), 5 - pKC5296KO plasmid (900 bp), 6 - Δ5296 genome DNA (1400 bp), 7 –1 Kb DNA Ladder.
